# Diagnostic yield, safety and therapeutic consequences of myocardial biopsy in clinically suspected fulminant myocarditis unweanable from mechanical circulatory support

**DOI:** 10.1186/s13613-023-01169-y

**Published:** 2023-08-31

**Authors:** Yann Marquet, Guillaume Hékimian, Guillaume Lebreton, Mathieu Kerneis, Philippe Rouvier, Pierre Bay, Alexis Mathian, Nicolas Bréchot, Juliette Chommeloux, Matthieu Petit, Melchior Gautier, Lucie Lefevre, Ouriel Saura, David Levy, Paul Quentric, Quentin Moyon, Sofia Ortuno, Matthieu Schmidt, Pascal Leprince, Charles-Edouard Luyt, Alain Combes, Marc Pineton de Chambrun

**Affiliations:** 1 Service de Médecine Intensive-Réanimation, Sorbonne Université, Assistance Publique-Hôpitaux de Paris (APHP), Hôpital La Pitié–Salpêtrière, 47–83, Boulevard de l’Hôpital, 75651 Paris Cedex, France; 2 Institut de Cardiométabolisme et Nutrition (ICAN), Sorbonne Université, INSERM, UMRS_1166-ICAN, 75013 Paris, France; 3 Service de Chirurgie Cardio-Thoracique, Sorbonne Université, Assistance Publique-Hôpitaux de Paris (APHP), Hôpital La Pitié–Salpêtrière, Paris, France; 4 ACTION Study Group, Département de Cardiologie, Sorbonne Université, APHP, Hôpital La Pitié-Salpêtrière, Paris, France; 5 Service d’Anatomopathologie, Sorbonne Université, APHP, Hôpital La Pitié–Salpêtrière, Paris, France; 6 AP-HP, Hôpitaux Universitaires Henri Mondor, DMU Médecine, Service de Médecine Intensive Réanimation and UPEC (Université Paris Est), INSERM, Unité U955, Équipe 18, 94010 Créteil, France; 7 Service de Médecine Interne 2, Centre de Référence National Lupus Systémique, Syndrome des Anticorps Anti-Phospholipides et Autres Maladies Auto-Immunes Systémiques Rares, Sorbonne Université, APHP, Hôpital La Pitié–Salpêtrière, Institut E3M, Paris, France; 8 Sorbonne Université, Inserm, Centre d’Immunologie et des Maladies Infectieuses (CIMI-Paris), Paris, France

**Keywords:** Fulminant myocarditis, Mechanical circulatory support, Endomyocardial biopsy, Diagnostic yield, Extracorporeal membrane oxygenation, Clinically suspected myocarditis

## Abstract

**Background:**

Fulminant myocarditis is a rare and severe disease whose definite and etiological diagnoses rely on pathological examination. Albeit, myocardial biopsy can be associated with significant morbidity and mortality, its therapeutic consequences are unclear. We conducted a study to determine the diagnostic yield, the safety and the therapeutic consequences of myocardial biopsy in patients with fulminant clinically suspected myocarditis unweanable from mechanical circulatory support (MCS).

**Methods:**

Monocenter, retrospective, observational cohort study in a 26-bed French tertiary ICU between January 2002 and February 2019. Inclusion of all fulminant clinically suspected myocarditis patients undergoing in-ICU myocardial biopsy while being on MCS. The primary endpoint was the proportion of patients classified as definite myocarditis using Bonaca criteria before and after including myocardial biopsy results.

**Results:**

Forty-seven patients (median age 41 [30–47], female 53%) were included: 55% died before hospital discharge, 34% could be bridged-to-recovery and 15% bridged-to-transplant. Myocardial biopsy was endomyocardial or surgical in 36% and 64% cases respectively. Tamponade requiring emergency pericardiocentesis occurred in 29% patients after endomyocardial biopsy. After adding the biopsy results in the Bonaca classification algorithm the percentage of definite myocarditis raised from 13 to 55% (p < 0.0001). The rate of biopsy-related treatments modifications was 13%, leading to patients’ recovery in only 4% patients.

**Conclusions:**

In clinically suspected myocarditis unweanable from MCS, myocardial biopsy increased the rate of definite myocarditis but was associated with a low rate of treatment modification and a significant proportion of adverse events. We believe the benefit/risk ratio of myocardial biopsy should be more carefully weighted in these frail and selected patients than suggested by actual guidelines. Further prospective studies are now needed to determine its value in patients under MCS.

**Graphical Abstract:**

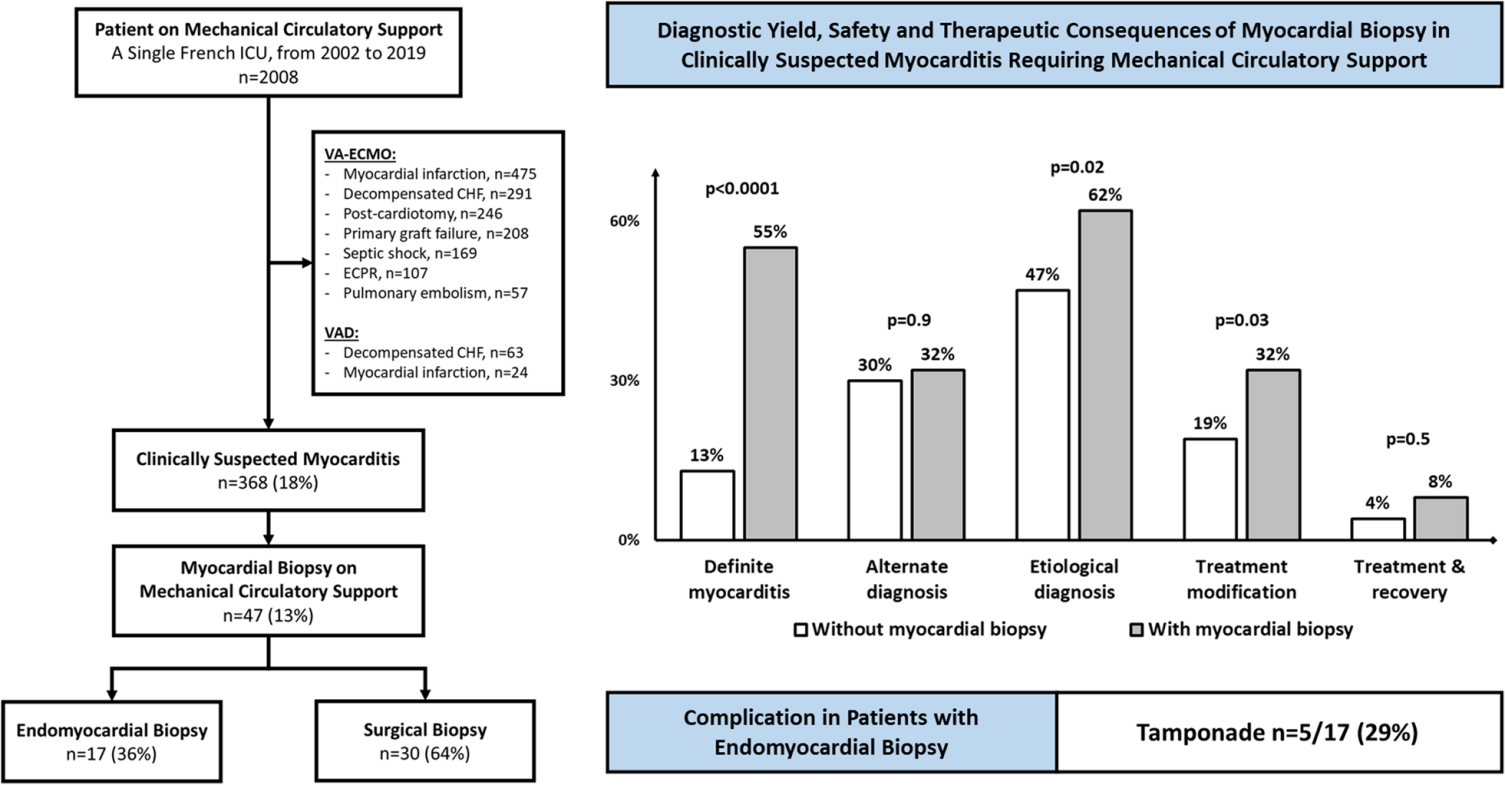

**Supplementary Information:**

The online version contains supplementary material available at 10.1186/s13613-023-01169-y.

## Introduction

Fulminant myocarditis is a rare and severe disease either due to a viral, bacterial or an immune-disease-related (giant-cell myocarditis, eosinophilic myocarditis, connective-tissue disease…) cardiac aggressions. Several causes of myocarditis can improve with specific treatments [1]. Definite and etiological diagnoses of myocarditis classically rely on myocardial pathological examination. Previous guidelines suggested that myocardial biopsies should be considered in every patient with clinically suspected myocarditis [2, 3]. More recently, the American and European guidelines recommended that biopsy should especially be considered among patients presenting with a severe form of myocarditis i.e.: cardiogenic shock, second degree atrioventricular block or higher, sustained or symptomatic ventricular tachycardia, or unresponsive to guideline-based medical management within 1–2 weeks [1, 4, 5].

The rate of myocardial biopsy performed among patients with suspected myocarditis is very heterogeneous worldwide, based on local expertise and habits [5]. It is low (< 4%) and even lowering in the USA, probably due to the advances in non-invasive diagnostic techniques [6]. The rates of biopsy ranged from 0 to 31% in three recent series of COVID-19 infection or vaccine-related clinically suspected myocarditis [7–9]. Moreover, physicians are sometimes reluctant to conduct myocardial biopsy as it can be associated with significant morbidity and mortality while its therapeutic consequences may appear unclear, especially among patients with refractory cardiogenic shock. In our experience, myocardial biopsy has low diagnostic yield, therapeutical consequences and significant morbidity in this setting, while the level of evidence supporting its use is low.

The objectives of this study were to determine the safety, the diagnostic yield and the therapeutic consequences of myocardial biopsy in patients with fulminant clinically suspected myocarditis unweanable from MCS.

## Materials and methods

### Patients

We retrospectively included all fulminant clinically suspected myocarditis (as defined by the ESC consensus statement [3]) patients from our 26-bed intensive care unit (ICU) who underwent a myocardial biopsy while on MCS between January 2002 and January 2019. Patients were included whether they underwent endomyocardial (EMB) or surgical biopsy (for instance during extracorporeal membrane oxygenation (ECMO) centralization or ventricle assisting device (VAD) implantation). They could not be included if the biopsy had been taken before ICU admission, after discharge or in-ICU not on MCS.

### Myocarditis noninvasive diagnosis work-up

Every fulminant clinically suspected myocarditis patient admitted to our ICU underwent a systematic noninvasive diagnostic work-up including laboratory analyses, imaging examinations and low-risk pathological examinations (i.e. salivary accessory gland biopsy). This work-up significantly changed during the time span of the study. Our comprehensive work-up and the corresponding investigated diseases, as well as the frequencies of each examen performed in the study population are reported in Additional file 1: Table S1. Cardiac magnetic resonance (CMR) was performed when possible before MCS implantation, or after weaning in recovering patients as it can provide a retrospective diagnosis of myocarditis. We used Lake-Louise criteria for the CMR diagnosis of myocarditis [10].

### Myocardial biopsy protocol

In our institution, patients with fulminant clinically suspected myocarditis do not systematically undergo myocardial biopsy. Myocardial biopsy is usually conducted in patient with unrecovering myocardial function after multidisciplinary discussion between intensivists, cardiologists, cardiac surgeons and internal medicine physicians. As a consequence, a significant number of our biopsies are taken on the occasion a central MCS implantation, performed in a bridging strategy of these unrecovering patients. EMB were performed in the catheterization laboratory under fluoroscopy. Right ventricle or left ventricle biopsy were taken on a case-to-case basis. For right ventricle EMB, the right internal jugular vein or femoral vein were used as the percutaneous access site while it was the femoral or radial artery for left ventricle EMB. Left or right ventricle surgical biopsies were taken on the occasion of open-heart surgery under visual guidance and followed by surgical hemostasis. Myocardial samples were fixed in 10% buffered formalin at room temperature for light microscopic examination. Multiple and numbered haematoxylin–eosin section examination were performed and when appropriate additional histochemical, histomorphologic, and immunohistochemical stains where analysed. Moreover, microbiological investigations were conducted on myocardial samples: bacterial cultures, viral polymerase chain-reactions and more recently metagenomic next generation sequencing. All biopsies were retrospectively reviewed by an expert cardiac pathologist for the purpose of this study.

### Data collection

The following informations were collected on standardized forms: epidemiological parameters; acute heart failure clinical, biological and therapeutic history; clinical manifestations; laboratory findings; MCS type, indication and complication(s); in-ICU organ-support treatments; noninvasive myocarditis work-up results; myocardial biopsy characteristics, results and complications; biopsy results; treatments introduced in the ICU; ECMO-weaning status; bridge-to-transplantation or ventricular assist device (LVAD); ICU complications; vital status, transplantation status at ICU and hospital discharges and at last follow-up.

### Outcome measures

The primary endpoint was the proportion of patients classified as definite myocarditis using Bonaca classification criteria [11] before then after myocardial biopsy results. We used the Bonaca cardio-oncology myocarditis classification criteria instead of ESC/AHA guidelines as our primary endpoint for their more inclusive nature and their high real-life applicability [11]. The secondary endpoints included: complications following myocardial biopsy; the proportion of: definite myocarditis diagnosis; etiological diagnosis; treatment changes and treatment changes leading to myocardial recovery using biopsy-based and noninvasive diagnosis work-up. As complications following surgical biopsies, performed on the occasion of central MCS implantation, may not be related to the biopsy itself, we separately analyzed the adverse events of surgical and endomyocardial biopsies.

### Statistical analyses

Results for categorical variables, expressed as number (%), were compared with χ^2^ or the exact test of Fischer; those for continuous variables, expressed as median [25th–75th percentile interquartile range (IQR)], were compared using Wilcoxon’s rank test. Categorical variables of invasive and noninvasive diagnosis work-up were compared using the test of McNemar. Statistical significance was defined as p ≤ 0.05. Analyses were computed with IBM SPSS Statistics v22.0 software (IBM Corp, Armonk, NY).

### Ethical considerations

The database is registered with the “*Commission Nationale de l’Informatique et des Libertés*” (2217847v0). In accordance with the ethical standards of our hospital’s institutional review board, the Committee for the Protection of Human Subjects, and French law, written informed consent was not needed for demographic, physiological and hospital-outcome data analyses because this observational study did not modify existing diagnostic or therapeutic strategies; however, patients were informed of their inclusion in the study.

## Results

### Characteristics, in-ICU organ failures and main outcomes

**Fig. 1:**
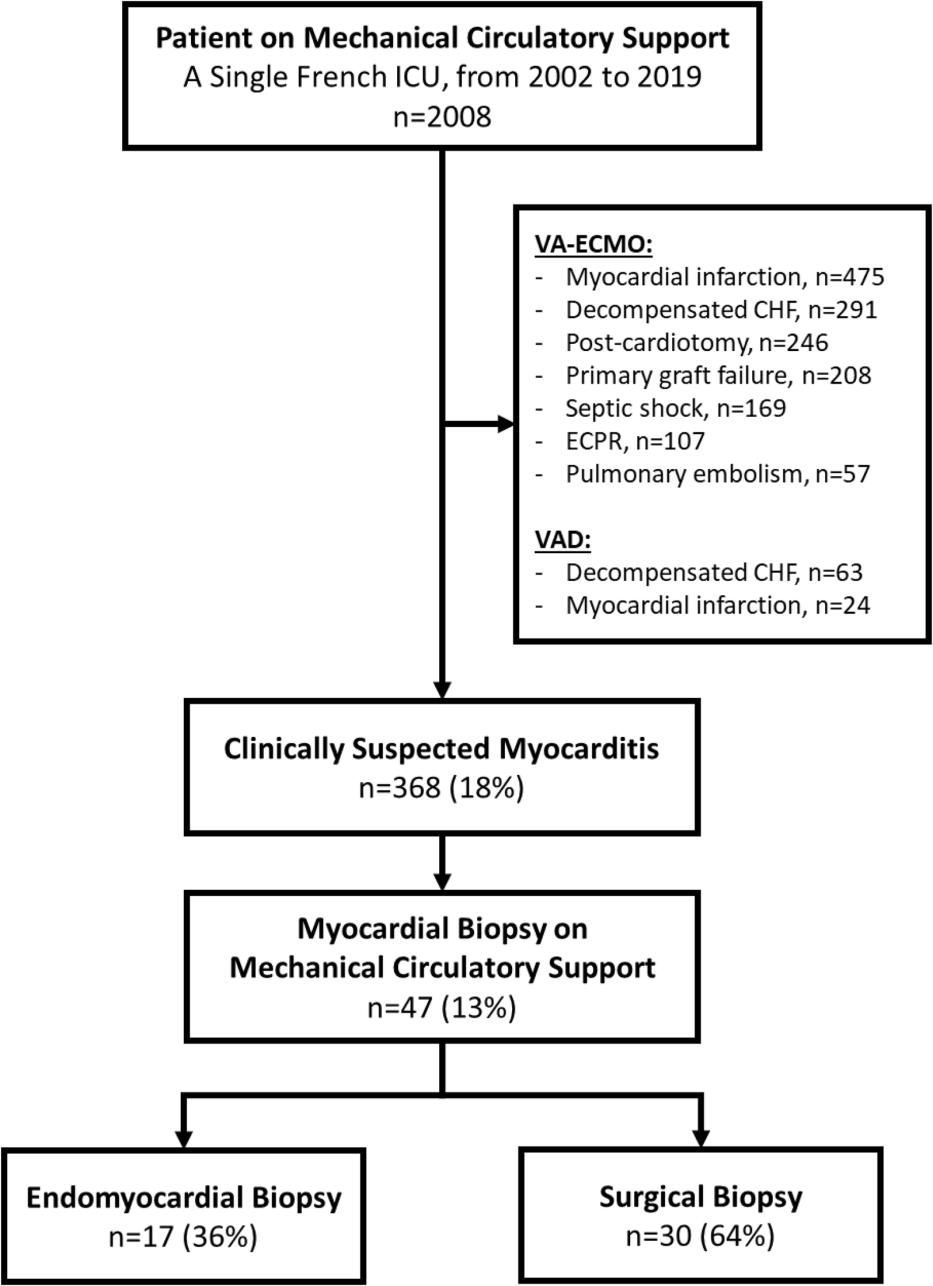
Flow-chart of the study. *ICU* intensive care unit, *VA-ECMO* venoarterial-extracorporeal membrane oxygenation, *CHF* chronic heart failure, *ECPR* extracorporeal cardiopulmonary circulation, *VAD* ventricle assist device

From 2002 to 2019, 2008 patients received MCS, 368 met criteria for clinically-suspected myocarditis and 47 (median age at admission 41 [30–47], female 53%) underwent a myocardial biopsy while on MCS and were recruited in the study (Fig. 1 and Table 1). Their main reported past medical history were: cardiovascular comorbidities 19%, autoimmune or inflammatory disease 17%, allergy 15% and cancer 8%. Median day-0 Simplified Acute Physiology Score II (SAPS II) score was 45 [33–65] and Sequential Organ Failure Assessment (SOFA) score was 10 [6–15]. In-ICU circulatory failure treatments included: inotropes/vasopressors 98%, venoarterial extracorporeal membrane oxygenation 89% (median duration 16 [9–34] days) and ventricle assist devices 32% (median duration 23 [17–219] days). Mechanical ventilation and renal replacement therapy were needed in 94% and 51% patients respectively. Twenty-six (55%) patients died before hospital discharge, 16 (34%) could be bridged-to-recovery and 7 (15%) bridged-to-transplant. Twelve (25%) patients recovered a left ventricle ejection fraction > 50% after MCS weaning.

**Table 1:** Characteristics, In-ICU organ failures and outcomes of the study population

Variables	n = 47
Female	25 (53)
Body mass index, kg/m^2^	23.9 [21.4–27.8]
Age at admission, years	41 [30–47]
Past medical history	
Cardiovascular comorbidities	9 (19)
Autoimmune/autoinflammatory disease	8 (17)
Allergic disease	7 (15)
Cancer	4 (8)
Myocarditis	2 (4)
Day-0 ICU scores	
Charlson comorbidity index	0 [0–1]
Day-0 SAPS II score	45 [33–65]
Day-0 SOFA score	10 [6–15]
In-ICU organ failures	
Circulatory failure	
Highest arterial lactate value, mmol/L	7 [4–13]
Inotropes/vasopressors	46 (98)
Venoarterial extracorporeal membrane oxygenation (VA-ECMO)	42 (89)
Femoro-femoral cannulation	39/42 (93)
Secondary centralization	26/42 (62)
Time on VA-ECMO, days	16 [9–34]
Left ventricular venting under VA-ECMO	25 (53)
Intra-aortic balloon counterpulsation	23 (49)
IMPELLA^®^ device	4 (8)
Ventricular assist device (VAD)	15 (32)
Left ventricular assist device	8/15 (53)
Bi-ventricular assist device	7/15 (47)
Time on VAD, days	23 [17–219]
Respiratory failure	
Mechanical ventilation	44 (94)
Time on mechanical ventilation, days	21 [10–36]
Renal failure	
Renal replacement therapy (RRT)	24 (51)
Time on RRT, days	14 [2–18]
Highest in-ICU serum creatinine value, µmol/L	161 [102–268]
Hematological failure	
Lowest in-ICU platelet count, G/L	41 [24–71]
Liver failure	
Highest in-ICU ALT value, UI/L	574 [288–1736]
Highest in-ICU bilirubin value, µmol/L	61 [28–134]
Lowest in-ICU prothrombin time, %	39 [27–47]
Outcomes	
Duration of follow-up, months	3 [1–87]
Time in ICU, days	22 [15–41]
Time in hospital, days	38 [22–74]
Bridge-to-recovery	16 (34)
LVEF recovery > 50%	12 (25)
Bridge-to-transplant	7 (15)
In-hospital mortality	26 (55)

Continuous variables are expressed as median [interquartile range 25–75]; categorical variables are expressed as No. (%)

*ICU* intensive care unit, *SAPS II* simplified acute physiology score, *SOFA* sequential organ failure assessment, *VA-ECMO* venoarterial extracorporeal membrane oxygenation, *VAD* ventricular assist device, *RRT* renal replacement therapy, *ALT* alanine transaminase

### Cardiovascular diseases findings

Flu-like illness, acute chest pain, and cardiac arrest were reported in 75%, 40% and 32% patients respectively (Table 2). The frequencies of electrocardiographic abnormalities were: conduction disorders 47%, rhythm disorders 47%, ST-segment elevation 36% and depression 11%. Troponin was elevated in 98% cases with a median value 110 [39–325] fold over upper limit of normal value (ULN). Coronary angiography was performed in 31 patients without disclosing obstructive coronary artery in any case. Median lowest in-ICU left ventricle ejection fraction (LVEF) value was 5% with left, right ventricle involvement and pericardial effusion reported in 100%, 60% and 49% respectively. CMR was available in only 9 patients, owing the time span of the study and frequent contraindications; with positive Lake-Louise criteria in 6 patients.

**Table 2:** Cardiovascular findings in the 47 patients with fulminant clinically suspected myocarditis on MCS

Variables	n = 47
Time from symptoms onset to hospital admission, days	3 [0–14]
Myocarditis syndrome	47 (100)
Acute chest pain	19 (40)
Flu-like illness	35 (74)
Cardiogenic shock	47 (100)
Cardiac arrest	15 (32)
Out-of-hospital	1 (2)
Shockable rhythm	3/15 (20)
No-flow duration, min	0 [0–0]
Low-flow duration, min	10 [2–22]
Elevated biomarkers	46 (98)
Troponin highest value, fold over ULN	110 [39–325]
Electrocardiographic anomalies	40 (85)
ST-segment depression	5 (11)
ST-segment elevation	17 (36)
Rhythm disorders	22 (47)
Supraventricular	13/22 (59)
Ventricular	9/22 (41)
Conduction disorders	22 (47)
Complete heart-block	7/22 (32)
Echocardiographic anomalies	47 (100)
LVEF lowest value, %	5 [5–10]
LVOT VTI lowest value, %	5 [0–6]
Left ventricle involvement	47 (100)
Right ventricle involvement	28 (62)
Pericardial effusion	23 (49)
LVEDD, mm	55 [52–60]
Coronary angiography	31 (66)
No coronary obstruction	31 (100)
CMR myocarditis pattern	6/9 (67)
Bonaca classification^a^ after noninvasive work-up	
Definite myocarditis	6 (13)
Probable myocarditis	41 (87)

Continuous variables are expressed as median [interquartile range 25–75]; categorical variables are expressed as No. (%)

*MCS* mechanical circulatory support, *ULN* upper limit of normal value, *LVEF* left ventricle ejection fraction, *LVOT VTI* left ventricular outflow tract velocity–time integral, *LVEDD* left ventricle end-diastolic diameter, *CMR* cardiac magnetic resonance

^a^Bonaca classification is available in [11]

### Myocardial biopsies characteristics

Seventeen (36%) patients had an EMB and 30 (64%) a surgical biopsy (Table 3). On biopsy-day, organ failure treatments were: inotropes/vasopressors 96%, VA-ECMO 89%, mechanical ventilation 74%, and renal replacement therapy 30% and VAD 11%. The time from first MCS to biopsy was 5 days. Before biopsy, anticoagulant and antiplatelet agent were administrated in 87% and 2% patients, respectively. Biopsy-day median platelet count was 95 [64–160] and 25% patients received platelet transfusion before biopsy. After EMB, 5/17 (29%) had a tamponade requiring emergency pericardiocentesis (percutaneous in all with additional surgical revision in 2 cases). One patient died as a direct consequence of the EMB. Surgical biopsies were associated with: need for surgical revision 7/30 (23%) and tamponade 3/30 (10%). Aside of tamponade, indication for surgical revision were surgical-site infection or device dysfunction.

**Table 3:** Myocardial biopsies characteristics and complications

Variables	n = 47
Biopsy characteristics	
Time from ICU admission to biopsy, days	6 [3–11]
Endomyocardial biopsy	8 [5–11]
Surgical biopsy	5 [2–10]
Time from first MCS to biopsy, days	5 [2–10]
Endomyocardial biopsy	17 (36)
Surgical biopsy	30 (64)
VAD implantation	12/30 (40)
VA-ECMO centralization	16/30 (53)
Biopsy site^b^	n = 28
Left ventricle^c^	19/28 (68)
Right ventricle^d^	7/28 (25)
Both ventricles^e^	2/28 (7)
Organ-failures on biopsy-day	
Inotropes/vasopressors	45 (96)
VA-ECMO	42 (89)
Intra-aortic balloon counterpulsation	22 (47)
IMPELLA^®^ device	2 (4)
VAD surgery	5 (11)
Mechanical ventilation	35 (74)
Renal replacement therapy	14 (30)
Hemostasis findings on biopsy-day	
Antiplatelet agent	2 (4)
No interruption before biopsy	2/2 (100)
Anticoagulation	41 (87)
Unfractionned heparin	40/41 (98)
Dose, 10^3^ UI/day	15 [10–24]
Interruption before biopsy	38/41 (93)
Duration of interruption before biopsy, hours	1 [1–1]
Duration of interruption after biopsy, hours	2 [0.7–24]
Laboratory value before biopsy	
Platelet count, G/L	95 [64–160]
APPT ratio	1.5 [1.2–1.8]
Prothrombin time, %	65 [53–75]
Transfusion before biopsy	
Platelets	12 (25)
Fresh frozen plasma	7 (15)
Complication after biopsy	
Endomyocardial biopsy	*n* = *17*
Tamponade	5 (29)
Bedside pericardial drainage	5 (100)
Surgical drainage	2 (40)
Surgical biopsy	*n* = *30*
Tamponade	3 (10)
Surgical revision^a^	7 (23)
Biopsy-related mortality	1 (6)

Continuous variables are expressed as median [interquartile range 25–75]; categorical variables are expressed as No. (%)

*ICU* intensive care unit, *VA-ECMO* venoarterial extracorporeal membrane oxygenation, *VAD* ventricle assist device, *APPT* activated partial thromboplastin clotting time

^a^For any reason

^b^Surgical biopsy n = 19, endomyocardial biopsy n = 9

^c^Surgical biopsy n = 13, endomyocardial biopsy n = 6

^d^Surgical biopsy n = 4, endomyocardial biopsy n = 3

^e^Surgical biopsy n = 2, endomyocardial biopsy n = 0

### Diagnostic yield and therapeutical consequences

**Fig. 2:**
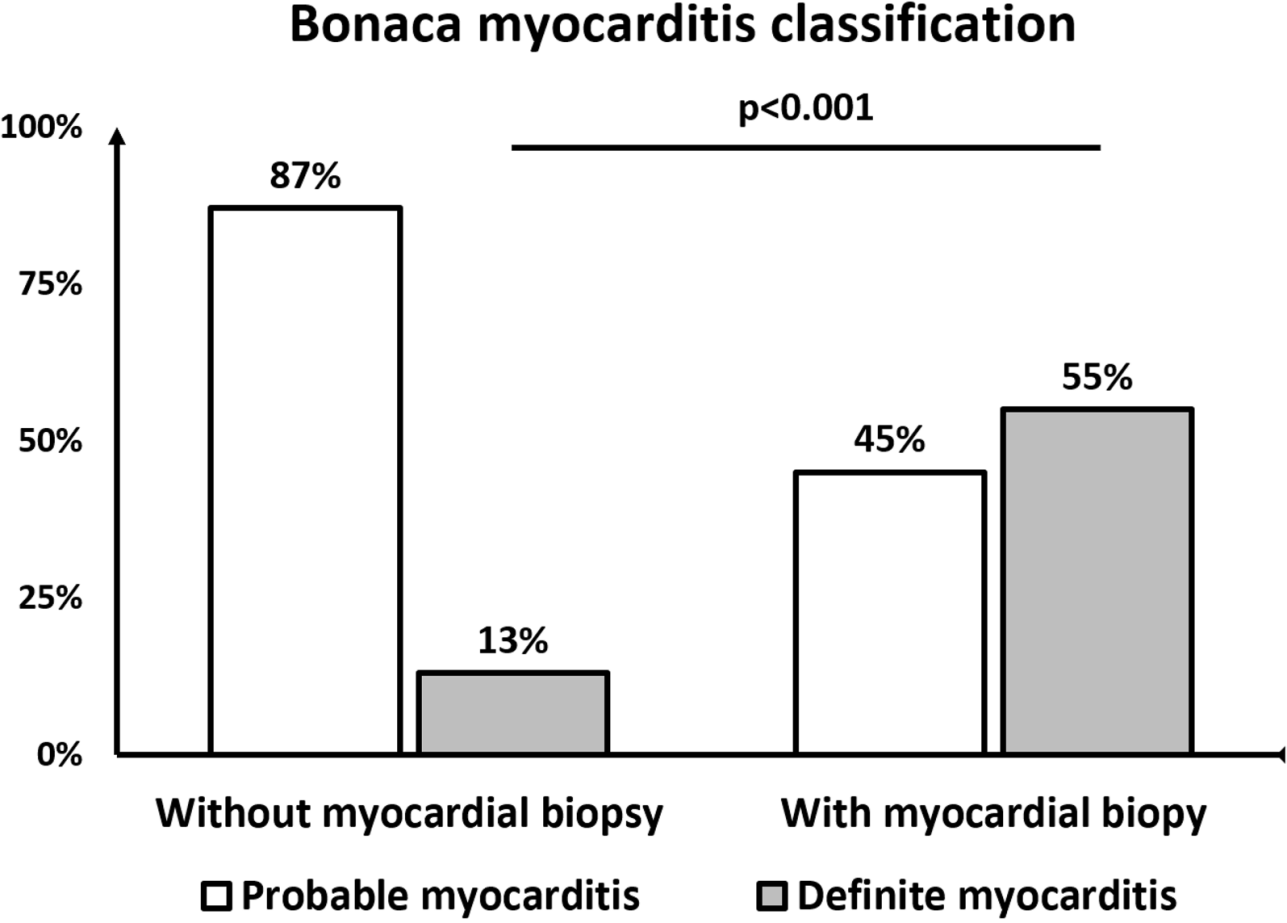
Bonaca myocarditis classification before and after myocardial biopsy results. Results of the test of McNemar between rates of definite diagnosis before and after biopsy results: p < 0.0001

**Fig. 3:**
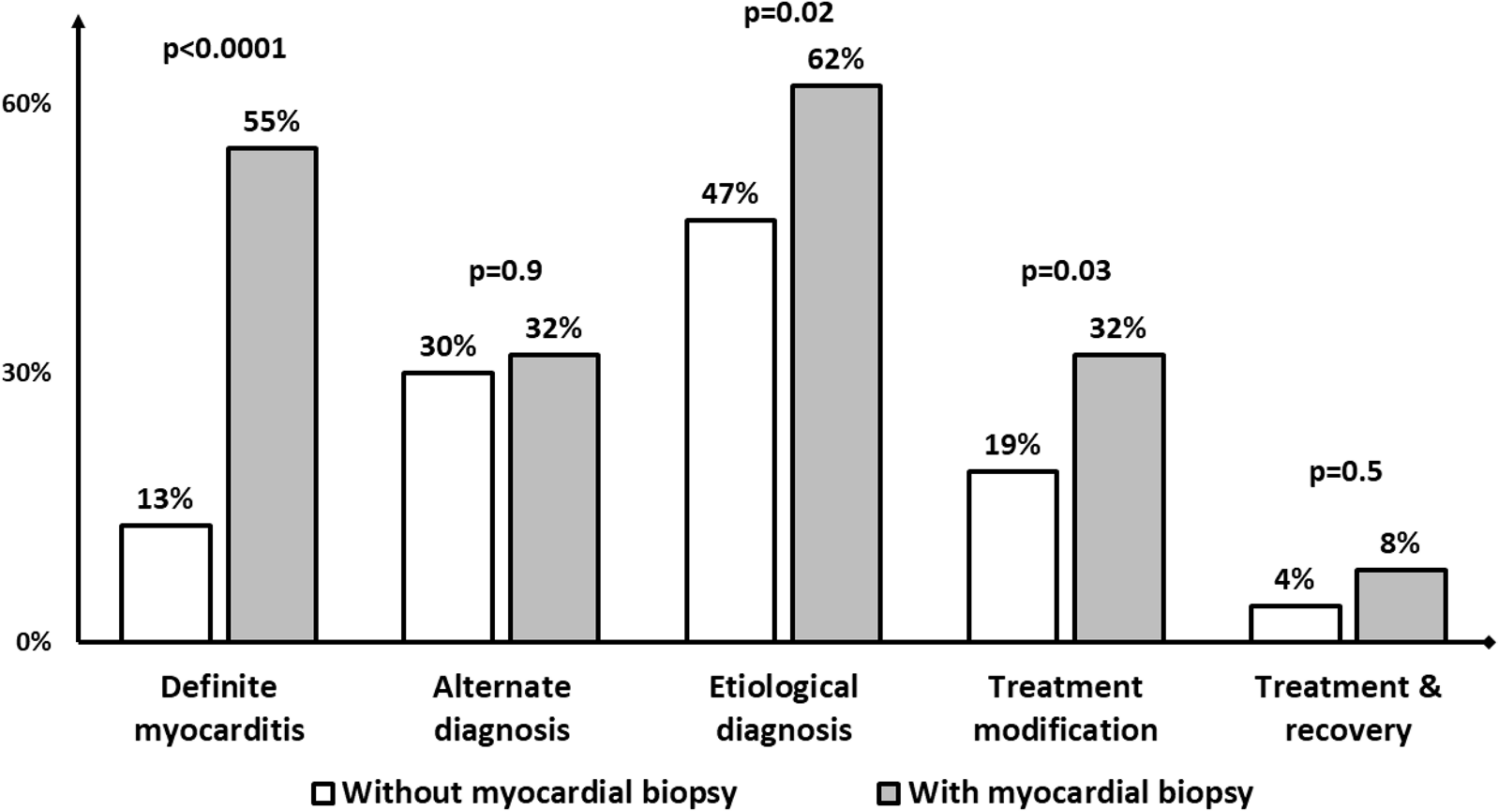
Myocarditis, alternate, etiological diagnosis, treatment changes and success in with noninvasive and biopsy-based work-up. p-value represents the results of the test of McNemar between noninvasive and invasive diagnosis work-up. Grey bars represent the diagnostic yield of the integration of both noninvasive and biopsy-based work-up

The noninvasive and the biopsy-based work-up disclosed a diagnosis of myocarditis, an alternate diagnosis and an etiological diagnosis to the cardiac disease in 13%/51%, 30%/4% and 47%/21% cases respectively (Table 4). After adding the results of the biopsy in the Bonaca classification algorithm the percentage of definite myocarditis raised from 13 to 55% (p < 0.0001) (Fig. 2). Similarly, the rates of etiological diagnoses (47 to 62%, p = 0.02) and therapeutic modifications (19 to 32%, p = 0.03) significantly increased after biopsy results while the frequencies of alternate diagnoses (30 to 32%, p = 0.9) and therapeutic modifications leading to recovery (4 to 8%, p = 0.5) were non significantly improved (Fig. 3). Additional file 1: Figure S1 reports the detailed results of noninvasive and biopsy-based diagnosis work-up.

**Table 4:** Diagnosis and therapeutic consequences of noninvasive and biopsy-based diagnostic work-up

Variables	n = 47
Noninvasive diagnosis work-up	
Diagnostic yield	
Myocarditis	6 (13)
Alternate diagnosis	14 (30)
Decompensated dilated cardiomyopathy	11 (23)
MINOCA	2 (4)
Acute cardiomyopathy	1 (2)
Etiological diagnosis	22 (47)
Eosinophilic myocarditis	5 (11)
Connective tissue disease-related myocarditis^a^	4 (8)
Chemotherapy-induced cardiomyopathy	3 (6)
Arrhythmic cardiomyopathy	2 (4)
Bacteria-induced cardiomyopathy^b^	2 (4)
Post-partum cardiomyopathy	2 (4)
RNA polymerase III autoantibodies-associated myocarditis	1 (2)
Others^c^	3 (6)
Therapeutic modifications	9 (19)
Immunosuppressant/immunomodulatory drugs^d^	6 (13)
Antiviral therapy/antibiotics	2 (4)
Early cardiac transplantation	1 (2)
Biopsy-based diagnosis work-up	
Diagnostic yield	
Myocarditis	24 (51)
Lymphocytic myocarditis	13 (28)
Eosinophilic myocarditis	5 (11)
Giant-cell myocarditis	2 (4)
Borderline myocarditis	2 (4)
Alternate diagnosis	2 (4)
Myocardial infarction	1 (2)
Inherited cardiomyopathy	1 (2)
Absence of diagnosis	18 (38)
No myocardial sample	5 (11)
Virus-positive sample	7/39 (18)
Epstein-Barr Virus	4/39 (10)
Parvovirus B19	3/39 (8)
Etiological diagnosis	10 (21)
Eosinophilic myocarditis	5 (11)
Giant-cell myocarditis	2 (4)
Group B streptococcus myocarditis	1 (2)
Enterovirus-related myocarditis	1 (2)
EBV-related myocarditis	1 (2)
Therapeutic modifications	6 (13)
Immunosuppressant/immunomodulatory drugs^e^	5 (11)
Antiviral therapy/antibiotics	1 (2)
Bonaca classification after biopsy results	
Definite myocarditis	26 (55)
Probable myocarditis	21 (45)
Myocardial recovery under treatment	4 (8)
Noninvasive work-up driven treatment^f^	2 (4)
Biopsy driven treatment^g^	2 (4)

Continuous variables are expressed as median [interquartile range]; categorical variables are expressed as No. (%)

*MINOCA* myocardial infarction without obstructive coronary artery, *RNA* ribonucleic acid, *EBV* Epstein-Barr virus

^a^Systemic sclerosis n = 1, idiopathic inflammatory myositis n = 1, catastrophic antiphospholipid syndrome n = 1, adult-onset Still’s disease n = 1

^b^Lyme’s disease n = 1, B group Streptococcus

^c^Amniotic embolism n = 1, viral myocarditis n = 1, decompensated alcoholic cardiomyopathy n = 1

^d^Corticosteroids alone n = 3, intravenous immunoglobulins alone n = 1, corticosteroids and intravenous immunoglobulins n = 1, anticoagulation, corticosteroids and plasma exchange/intravenous immunoglobulins (triple therapy) n = 1

^e^Aciclovir n = 1, corticosteroids n = 1, corticosteroids, thymoglobulins and mycophenolate mofetil n = 1

^f^Antibiotics for Lyme’s disease n = 1, corticosteroids for eosinophilic myocarditis n = 1

^g^Corticosteroids for eosinophilic myocarditis n = 2

## Discussion

This study reports the risk and benefit of EMB in a monocentric cohort of clinically suspected fulminant myocarditis unweanable of MCS. Our findings can be summarized as follow: 1. Myocardial biopsy in fulminant clinically suspected myocarditis unweanable from MCS patients increased the rate of definite myocarditis diagnosis according to Bonaca classification 2. It is associated with a low rate of treatment modification leading to myocardial recovery 3. Myocardial biopsy is associated with a significant and higher rate of adverse events than previously reported in non-severe acute myocarditis.

The diagnosis of fulminant myocarditis is one of the most challenging undertaking in modern cardiology. In this setting, all diagnosis breakthrough granted by cardiac imaging are unavailable given the hemodynamic instability or the need for mechanical circulatory support. For these cases, international guidelines recommend myocardial biopsy to be the cornerstone diagnostic investigation [1–3]. However, its safety, diagnosis yield and therapeutic consequences remain unclear in critically-ill patients requiring MCS, while many advances in immunology and microbiology allow swifter and new diagnostics.

The evaluation of EMB safety mainly arises from retrospective series, making the rate of complications uneasy to appraise. The reported rates of tamponade ranged between 0.3 and 0.9% in two recent large cohort from the USA and Germany [12, 13]. Most of these patients were non-severe patients from cardiology ward and the frequency of MCS is not reported. A recent study, focusing on patients requiring ECMO reported a significantly higher rate of tamponade, up to 18% of patients on ECMO [14]. Our higher frequency of hemopericardium underlines that safety of EMB in patients under MCS cannot be extrapolated from series mostly reporting non-severe patients. Further prospective studies are urgently need to determine the true frequency of severe EMB complications in patients under MCS. The American Heart Association (AHA) recently recognized fulminant clinically suspected myocarditis as a class I indication for EMB [1]. The benefit-risk ratio evaluation in this statement is based on the 2013 European Society of Cardiology (ESC) position paper on clinically suspected myocarditis [3] and the 2007 ESC/AHA statement on EMB [2], which almost exclusively proceed from series reporting the outcome of EMB in non-severe patients. There is an important knowledge gap on EMB safety in critically-ill patients, especially those on extracorporeal life supports. We believe the risks of EMB in this population may have been underestimated.

When considering myocarditis, the definition of “diagnostic yield” is a challenging concept. Indeed, several diagnosis categories can co-exist and sometimes merge into each other. For instance, lymphocytic and giant-cell myocarditis are two pathological diagnoses but only the latter constitutes a homogeneous clinical entity in which introducing an immunosuppressive regimen is recommended. Lymphocytic myocarditis can be encountered in various etiologies. Therefore, EMB diagnostic yield assessment should be multiparametric including: the diagnosis of myocarditis; the diagnosis of the cause and the identification of an alternate diagnosis. Bennett et al. found a 25% rate of diagnostic biopsies in 851 patients but up to 38% in a subgroup of acute unexplained heart failure with hemodynamic instability [12]. A similar rate was reported in the Kinderman et al. study (38%) including very few severe patients [15]. In the series by Van der Boon et al., EMB in new/acute heart failure resulted in a all-cause diagnosis in 52% and even 78% cases when considering patients requiring extracorporeal life supports [14].

The diagnostic yield of myocardial biopsy relies on the operator and anatomopathologist experiences and the yearly number of myocardial biopsy in this series is low. However, our center perform ≈ 1500 myocardial biopsies yearly, mainly in cardiac transplant recipient, confirming our position as an high case-volume and experienced center for myocardial biopsy. The consensual recommendations made by ESC/AHA suggest to take as soon as possible at least 4 specimens from different sites (right ventricle, left ventricle, interventricular septum) in experienced center with experienced operators, but there is a huge lack of evidence on significant clinical endpoint to support these recommendations.

Recent data suggest that cardiac CT scan with coronary and myocardial time could be helpful in ruling out coronary artery disease/myocardial infarction and making the positive diagnosis of myocarditis [16]. Yet, during most the time of the study, this technique was not routinely instituted for patients with fulminant myocarditis in our center. Coronary angiography should be only mandatory in fulminant clinically suspected myocarditis, especially those on MCS, but it was performed in only 66% of our patients. This result is however in line with the 56–63% rate of coronary angiography reported in three previous studies on fulminant myocarditis [7, 17, 18]. CMR has become the cornerstone for the diagnosis of acute myocarditis. When available, CMR should be used to guide EMB as myocarditis is frequently a patchy disease [10]. Albeit not available in fulminant myocarditis under MCS, CMR can be performed after weaning in recovering patient for a retrospective diagnosis of myocarditis. When CMR can not be performed, electroanatomic voltage mapping is an exciting and emerging technique to guide EMB [19].The evaluation of myocardial biopsy efficacy ends with its therapeutic consequences, especially those that will lead to recovery. In our cohort, patients underwent a biopsy while not recovering from their heart failure. This criterion constitutes an important selection bias but also removes from the treatment analysis all patients that spontaneously improved and could quickly be bridged-to-recovery. One of the only large series investigating this particular outcome, the study by Bennett et al. reported a low rate of “clinical course modification” associated with EMB: 27% in patients with hemodynamic instability [12]. We reported a lower rate of treatment modification, but we integrated the treatment changes granted by the noninvasive diagnostic work-up.

One could challenge the very need for myocardial biopsy. Any invasive procedure should only be considered when it might bring a therapeutic intervention the patient could benefit from. Achieving a diagnosis of definite myocarditis as compared to a probable [11] or clinically-suspected myocarditis [3] does not change patients’ management by itself. The vast majority of myocarditis spontaneously recovers. The prognostic value of EMB is well known, yet no study has shown that these differing outcomes reflect anything other than the natural severity of each disease [15, 17]. Most viral myocarditis can be proven with noninvasive testing [8], spontaneously recover and, to date, no specific treatment have shown to be effective. Recent evidence even suggests the virus may not be the cause of the disease itself [20]. What would be the point of a myocardial biopsy in a patient with COVID-19/flu-related myocarditis, where bronchoalveolar lavage or nasopharyngeal swab can easily and safely yield the diagnosis? The futility of therapeutic intervention in refractory fulminant giant-cell myocarditis has been reported [21]. We are on the verge of understanding that acute myocarditis can be the manifestation of inherited cardiomyopathy, an etiological diagnosis the biopsy can’t unveil and for which no specific therapeutic interventions under MCS have yet been recommended [22, 23]. Patient’s medical history and noninvasive tests are usually eloquent enough to achieve a diagnosis of connective-tissue disease, and the majority of eosinophilic myocarditis. In the recent ESC cardio-oncology guidelines, a definite diagnosis of immune checkpoint inhibitors-induced myocarditis can be adjudicated without EMB [24]. What is truly important is the identification of an etiological diagnosis leading to a clear therapeutic decision. However, no or few study reported the rate of etiological and even of alternate diagnosis of myocarditis. Our etiological diagnosis rate was disappointingly low, especially facing the results of the noninvasive diagnosis work-up. One of the main flaw in studies investigating EMB and in myocarditis guidelines is the frequent oblivion of the non-invasive myocarditis work-up. With the advances in biochemistry, microbiology and immunology, many diseases can now have a very quick and definite and noninvasive diagnosis.

We believe myocarditis diagnostic strategy should parallel the one used in interstitial lung disease (ILD). Either diseases can be acute or chronic, asymptomatic or fulminant and can be the expression of a very large number of diseases: toxic, infectious, genetic, metabolic, autoimmune, allergic… Open lung biopsy (OLB) and EMB both share low diagnostic yield and life-threatening complications. The benefit/risk ratio does not favor systematic OLB in every ILD patient. International ILD guidelines therefore placed OLB only at the end of the ILD diagnostic strategy. First, a multidisciplinary discussion evaluating patient’ medical history, clinical symptoms, lung imaging and a large non-invasive testing [25] will try to achieve a diagnosis or to offer a probabilistic treatment. When the multidisciplinary discussion fails, OLB can be considered. In this very little population, the benefit/risk ratio is much more acceptable. We believe placing EMB at the beginning of myocarditis diagnostic strategy is not only an unnecessary risk for many patients, but also diverts our attention to what is most important: the medical history, the clinical examination and the noninvasive assessment.

Our study has several strengths and limitations. It has a retrospective, monocentric, observational design but many patients with a rare disease could be included. Due to the inclusion period, a heterogeneity of management may have occurred. Nevertheless, the vast majority of the patients enrolled in this study were included during the last decade. As our population was highly selected (severe fulminant clinically suspected myocarditis unweanable from MCS) our results can not be generalized to an unselected population of patients admitted in ICU for a clinically suspected fulminant myocarditis. Both endomyocardial and surgical biopsy patients were included, while their accuracy, sampling bias and complications might not be similar. However, we reported separately their adverse event and this study reflects a real-life experience. To adjudicate biopsy consequences, we focused on the treatment changes following biopsy. Yet, we might have missed other type of treatment changes (drug interruption, decision to perform other tests, modification of bridging strategies…), limiting our conclusion on this finding.

## Conclusion

Myocardial biopsy use to investigate fulminant clinically suspected myocarditis unweanable from MCS increased the rate of definite myocarditis diagnosis according to Bonaca classification but was associated with a low rate of treatment modification leading to myocardial recovery and a significant rate of adverse events. We believe the benefit/risk ratio of myocardial biopsy should be more carefully weighted in these frail and selected patients than suggested by actual guidelines. Further prospective studies are now needed to determine the safety, diagnostic yield and therapeutic consequences of myocardial biopsy in fulminant myocarditis.

### Supplementary Information

**Additional file 1: Figure S1.** Flow-chart of the Diagnostic Yield and Therapeutical Consequences of Noninvasive and Biopsy-based Diagnosis Work-up. *APS* antiphospholipid syndrome, *AOSD* adult-onset Still disease, *MCS* mechanical circulatory support, *MINOCA* myocardial infarction with no obstructive coronary artery, *RNApol3* RNA-polymerase-III associated myocarditis. Non-invasive diagnosis panel: the 58% with no diagnosis are still considered as clinically-suspected myocarditis. Clinically suspected refers to the patients discharged with a diagnosis of “Clinically suspected myocarditis” including one having a diagnosis of adult-onset Still disease and one a diagnosis of hypereosinophilic syndrome with cardiac involvement. **Table S1.** Fulminant myocarditis noninvasive diagnostic work-up.

## Data Availability

None.

## Abbreviations

CMR: Cardiac magnetic resonance
ECMO: Extracorporeal membrane oxygenation
EMB: Endomyocardial biopsy
ICU: Intensive care unit
ILD: Interstitial lung disease
LVAD: Left ventricle assist device
LVEF: Left ventricle ejection fraction
MCS: Mechanical circulatory support
OLB: Open lung biopsy
SAPS: Simplified acute physiology score
SOFA: Sequential organ failure assessment
ULN: Upper limit of normal value
VAD: Ventricular assist device
