# Supplementary material for: Diagnostic yield, safety and therapeutic consequences of myocardial biopsy in clinically suspected fulminant myocarditis unweanable from mechanical circulatory support
Source: Ann Intensive Care. 2023 Aug 31;13:78. doi: 10.1186/s13613-023-01169-y (PMC10471530; doi:10.1186/s13613-023-01169-y)
Supplement: Supplementary file 1 — Additional file 1: Figure S1. Flow-chart of the Diagnostic Yield and Therapeutical Consequences of Noninvasive and Biopsy-based Diagnosis Work-up. APS antiphospholipid syndrome, AOSD adult-onset Still disease, MCS mechanical circulatory support, MINOCA myocardial infarction with no obstructive coronary artery, RNApol3 RNA-polymerase-III associated myocarditis. Non-invasive diagnosis panel: the 58% with no diagnosis are still considered as clinically-suspected myocarditis. Clinically suspected refers to the patients discharged with a diagnosis of “Clinically suspected myocarditis” including one having a diagnosis of adult-onset Still disease and one a diagnosis of hypereosinophilic syndrome with cardiac involvement. Table S1. Fulminant myocarditis noninvasive diagnostic work-up. [file 13613_2023_1169_MOESM1_ESM.docx]

**Figure S1**


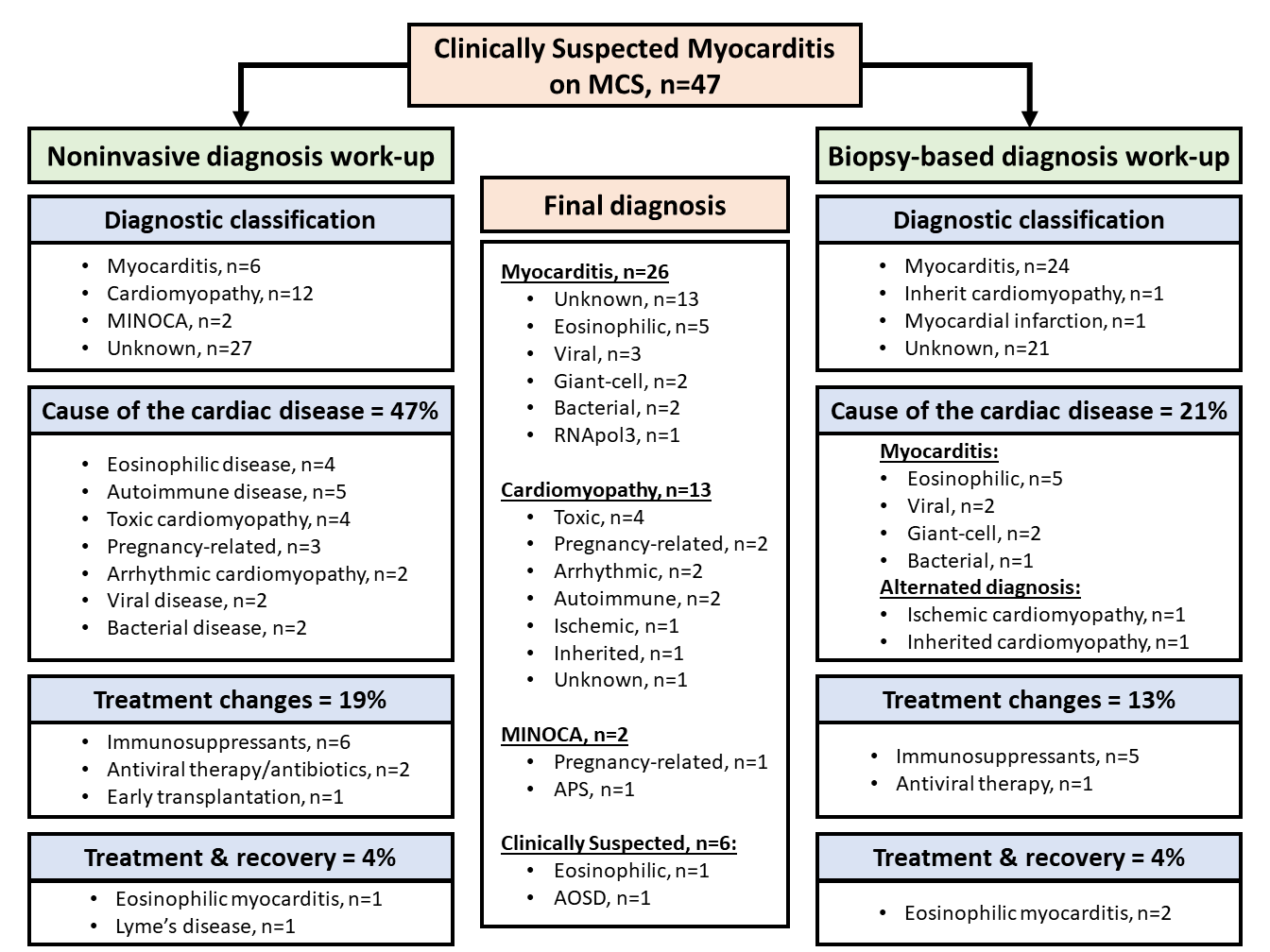


| **Table S1. Fulminant Myocarditis Noninvasive Diagnostic Work-up** | | |
| --- | --- | --- |
| **Variables** | **Disease investigated** | **Patients tested** |
| **Laboratory work-up** |  |  |
| **Biochemical work-up** |  |  |
| Angiotensin converting enzyme | Sarcoidosis | 20 (42) |
| TSH | Hypo or hyperthyroidism | 40 (85) |
| **Immunological work-up** |  |  |
| Antinuclear autoantibodies | Systemic lupus erythematosus  Idiopathic inflammatory myositis  Other connective tissue diseases | 47 (100) |
| Anti-DNA autoantibodies | Systemic lupus erythematosus | 42 (89) |
| Anti-ENA autoantibodies | Systemic lupus erythematosus  Idiopathic inflammatory myositis  Other connective tissue diseases | 39 (83) |
| Anti-tissue autoantibodies | Other connective tissue diseases | 7 (15) |
| Myositis immunodot | Idiopathic inflammatory myositis | 9 (19) |
| Systemic sclerosis immunodot | Systemic sclerosis and RNA polymerase III-associated myocarditis | 11 (23) |
| Anti-CCP autoantibodies | Rheumatoid arthritis | 4 (8) |
| ANCA | ANCA-associated vasculitis | 42 (89) |
| Antiphospholipid autoantibodies | Antiphospholipid syndrome | 13 (28) |
| Lupus anticoagulant | Antiphospholipid syndrome | 6 (13) |
| Complement fraction | Systemic lupus erythematosus | 41 (87) |
| Gammaglobulins | Sarcoidosis, connective tissue diseases, amyloidosis, common variable immunodeficiency, Clarkson’s disease | 30 (64) |
| Eosinophils count | Hyper eosinophilic syndrome and other eosinophilia-mediated diseases | 47 (100) |
| **Infectious work-up** |  |  |
| **Viruses screening** |  |  |
| **Viral serologies** |  |  |
| Parvovirus B19 | Parvovirus B19-related myocarditis | 18 (38) |
| Epstein-Barr Virus (EBV) | EBV-related myocarditis | 31 (66) |
| Cytomegalovirus (CMV) | CMV-related myocarditis | 31 (66) |
| Varicella Zoster Virus (VZV) | VZV-related myocarditis | 6 (13) |
| Herpes Simplex Virus (HSV) | HSV-related myocarditis | 11 (23) |
| Human Herpes Virus 8 (HHV8) | HHV8-related myocarditis, Castleman disease | 13 (28) |
| Human Immunodeficiency Virus (HIV) | HIV-related myocarditis | 41 (87) |
| Human T cell leukemia/lymphoma virus type 1 (HTLV-1) | HTLV-1-related myocarditis, Strongyloides Stercoralis hyperinfection | 23 (49) |
| Hepatitis A Virus (HAV) | HAV-related myocarditis | 28 (60) |
| Hepatitis B Virus (HBV) | HBV-related myocarditis, periarteritis nodosa | 43 (91) |
| Hepatitis C Virus (HCV) | HCV-related myocarditis, cryoglobulinemia-associated vasculitis | 43 (91) |
| **Viral PCR** |  |  |
| Respiratory viruses | Influenza-related myocarditis, coronavirus-related myocarditis, enterovirus-related myocarditis | 37 (79) |
| Parvovirus B19 | Parvovirus B19-related myocarditis | 25 (43) |
| Enteroviruses | Enterovirus-related myocarditis | 21 (45) |
| Epstein-Barr Virus (EBV) | EBV-related myocarditis | 36 (76) |
| Cytomegalovirus (CMV) | CMV-related myocarditis | 38 (81) |
| Varicella Zona Virus (VZV) | VZV-related myocarditis | 10 (21) |
| Herpes Simplex Virus (HSV) | HSV-related myocarditis | 31 (66) |
| Human Herpes Virus 6 (HHV6) | HHV6-related myocarditis | 33 (70) |
| Adenoviruses | Adenovirus-related myocarditis | 24 (51) |
| **Bacteria screening** |  |  |
| *Legionella pneumophila* | Legionella-related myocarditis | 32 (68) |
| *Chlamydia pneumonia* | Chlamydiae-related myocarditis | 32 (68) |
| *Mycoplasma pneumonia* | Mycoplasma-related myocarditis | 31 (66) |
| *Coxiella burnetii* | Q fever-related myocarditis | 32 (68) |
| Brucellosis | Brucellosis-related myocarditis | 4 (8) |
| Bartonella | Bartonella-related myocarditis | 2 (4) |
| *Borrelia burgdorferi* | Lyme’s disease-related myocarditis | 32 (68) |
| *Treponema pallidum* | Syphilis-related myocarditis | 27 (57) |
| Leptospirosis | Leptospirosis-related myocarditis | 9 (19) |
| **Toxoplasma gondii** | Toxoplasma-related myocarditis | 31 (66) |
| **Imaging work-up** |  |  |
| Coronary angiography | Myocardial infarction | 31 (66) |
| Full-body CT scan examination | Adrenal gland tumor or atrophy  Interstitial lung disease  Vein or artery stenosis or thrombosis  Lymphoma | 25 (53) |
| **Pathology work-up** |  |  |
| Accessory salivary gland biopsy | Sarcoidosis and amyloidosis | 3 (6) |
| Skin biopsy | Vasculitis, antiphospholipid syndrome | 0 (0) |
| Abbreviations: TSH, thyroid stimulating hormone; DNA, desoxyribonucleic acid; ENA, extractable nuclear antigen; CCP, cyclic citrullinated peptide; ANCA, antineutrophil cytoplasmic antibodies; PCR, polymerase chain reaction; CT, computed tomography. Continuous variables are expressed as median [interquartile range]; categorical variables are expressed as No. (%). | | |
|  |  | |
